# Optimizing Anti-Snake Venom Strategies for Hemotoxic Envenomation in Northern India: Clinical Outcomes and Regional Challenge

**DOI:** 10.7759/cureus.84090

**Published:** 2025-05-14

**Authors:** Ashutosh Ojha, Prakash Hadimani, Dawn Anthony, Vikas Raj, Sumit Bhasker, Mayank Mishra, Sharat Johri

**Affiliations:** 1 Department of General Medicine, Government Medical College, Maharashtra Post Graduate Institute of Medical Education and Research, Nashik, IND; 2 Department of General Medicine, Employees State Insurance Corporation (ESIC) Medical College and Hospital, Kalburagi, IND; 3 Department of General Medicine, Malankara Orthodox Syrian Church College of Nursing, Kolenchery, IND; 4 Department of Medicine, Pondicherry Institute of Medical Sciences, Kalapet, IND; 5 Department of Medicine and Geriatrics, Military Hospital, Jalandhar, IND; 6 Deparment of General Medicine, Military Hospital, Meerut, IND; 7 Department of Neurology, SRM Institute of Medical Sciences, Bareilly, IND

**Keywords:** anti-snake venom dosing, clinical outcomes, hemotoxic envenomation, northern india snakebite, varespladib therapy

## Abstract

Snakebite envenomation, especially from hemotoxic species such as *Daboia russelii* and *Echis carinatus*, remains a significant public health challenge in the northern Indian states of Punjab, Haryana, Uttar Pradesh, Uttarakhand, and Himachal Pradesh. Despite the availability of polyvalent anti-snake venom (ASV), inconsistent dosing strategies, delayed administration, and disparities in healthcare contribute to high morbidity and mortality rates. This review examines optimal ASV dosing protocols, clinical outcomes, and host-specific factors that influence the therapeutic efficacy in hemotoxic envenomation. Drawing from regional epidemiological data, toxicological insights, and clinical studies, the review underscores the influence of bite-to-needle intervals, ASV administration routes, and infrastructural readiness on patient survival. Notably, intravenous administration proves superior, while early intervention significantly reduces systemic complications. The study identifies key gaps in national guidelines, particularly the mismatch between regional venom variability and available ASV formulations. It also explores emerging alternatives like Varespladib and monoclonal antivenoms. Methodologically, the review adopts a narrative synthesis of peer-reviewed literature and policy frameworks. It concludes that standardizing ASV treatment based on regional evidence, enhancing healthcare capacity, and integrating public health education are essential to improving outcomes. The findings support the need for locally tailored, patient-centric treatment protocols and stronger public health systems to mitigate snakebite-related burdens.

## Introduction and background

Snakebite envenomation remains a critical public health emergency in India, particularly in rural areas, agricultural communities, and regions prone to monsoons. The World Health Organization (WHO) classifies snakebites as a neglected tropical disease. India alone accounts for nearly 50 percent of the estimated 138,000 annual global deaths and 400,000 disabilities caused by snakebites [1]. In the northern Indian states of Punjab, Haryana, Uttar Pradesh, Uttarakhand, and Himachal Pradesh, hemotoxic envenomation is predominantly caused by Russell’s viper (*Daboia russelii*) and the saw-scaled viper (*Echis carinatus*). These species release venom that induces coagulopathy (a disruption of normal blood clotting), internal bleeding, and acute kidney injury, conditions that can rapidly become life-threatening [1].

Timely administration of anti-snake venom (ASV), a serum-based therapeutic derived from immunized horses, is essential to reduce systemic damage. However, ASV usage varies substantially across regions. Delays in ASV delivery, inconsistent dosing practices, and the absence of standardized treatment protocols contribute to suboptimal outcomes. Additionally, serum sickness (a delayed immune response marked by fever, rash, and joint pain) and anaphylaxis (a severe allergic reaction) represent critical risks associated with ASV treatment [2]. Despite the availability of polyvalent ASV in India, uniform treatment protocols have not been established due to variable practices among healthcare providers. Differences in dose escalation strategies, severity of envenomation, and response time hinder the development of standardized guidelines. The venom of Russell’s viper spreads rapidly through the body, yet early administration of ASV significantly lowers the risk of complications and mortality [2]. The efficacy of ASV is heavily influenced by the timing of administration and the clinician’s ability to recognize early clinical signs [3].

Serum sickness and anaphylaxis frequently occur when ASV is administered without appropriate premedication or monitoring, particularly when large or rapid doses are given [4]. In resource-constrained areas, clinicians face uncertainty due to ambiguous guidelines and limited antivenom supplies [5]. Recent research has explored ASV pharmacokinetics through various delivery routes. While intravenous (IV) administration is generally preferred, evidence from India regarding differences between IV and intramuscular (IM) routes remains limited [6]. In rural regions such as Punjab and Haryana, the delay between snakebite and medical care is prolonged due to high environmental exposure and insufficient access to healthcare facilities. These delays reduce ASV efficacy and worsen clinical outcomes [7]. National initiatives, including the National Programme for Prevention and Control of Snakebite and the National Action Plan for Prevention and Control of Snakebite Envenoming (NAPSE), aim to address these challenges by improving surveillance, enhancing training, and streamlining ASV supply chains [8,9]. Nonetheless, implementation remains inconsistent across states, often overlooking regional venom variations and specific population characteristics.

**Figure 1:**
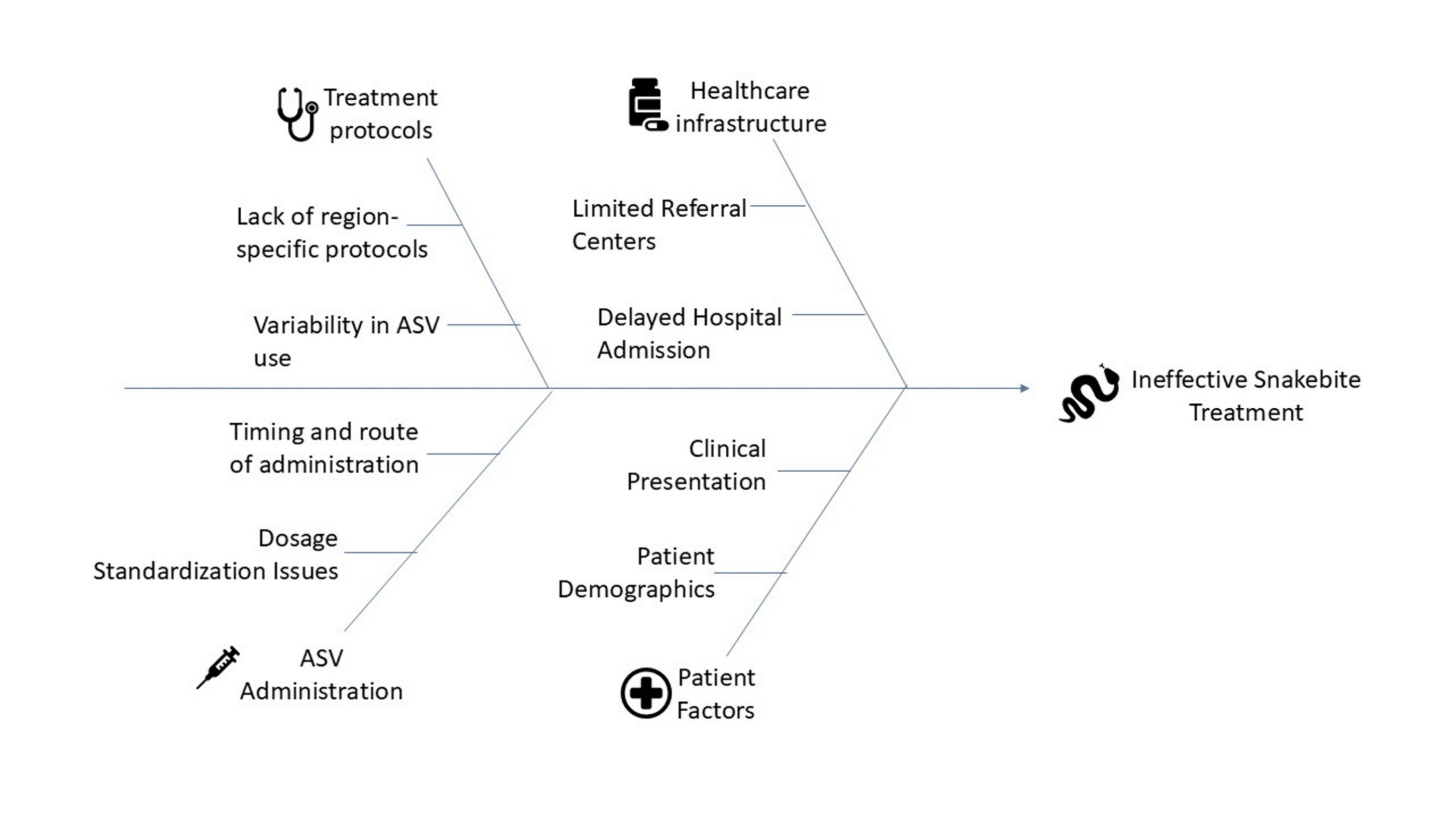
Major challenges contributing to ineffective snakebite treatment in India Image credit: Vikas Raj Figure created using Microsoft PowerPoint

While this review primarily focuses on hemotoxic snakebites, it acknowledges that neurotoxic cases also necessitate tailored treatment approaches. Studies reveal that outcomes for patients treated with polyvalent ASV vary according to clinical presentation and adherence to therapeutic protocols [10]. The evidence suggests that treatment should be individualized based on the species involved and patient-specific factors, rather than applying a universal standard. Emerging strategies include novel pharmacological interventions and refinement of antivenom therapies to enhance safety and efficacy [11]. Figure 1 shows major challenges contributing to ineffective snakebite treatment in India.

Objectives of the review

The review assesses the available evidence regarding ASV administration for treating hemotoxic envenomation cases in northern India. The review evaluates multiple doses of ASV alongside their timing and routes of administration to determine their effects on clinical results. The review analyzes a nationwide database alongside data from specific northern Indian regions to demonstrate why standardized protocols and dose thresholds need development according to local snake ecology. The analysis studies the relationships that develop between logistical difficulties and clinical operational practices, together with systemic healthcare limits, which impact the performance of ASV therapy and patient survival outcomes.

## Review

Epidemiological burden of hemotoxic snakebites in northern India

Snakebite envenomation continues as a major public health problem in India that mainly affects people living in rural agricultural areas. Seasonal and geographical conditions in the northern states of Punjab, Haryana, Uttar Pradesh, Uttarakhand, and Himachal Pradesh create elevated incidence rates. During the monsoon season, the peak period occurs when more people work in agricultural areas as snakes become more active. The risk of systemic complications after snakebites becomes more severe because of two poisonous species called *Daboia russelii *(Russell’s viper) and *Echis carinatus* (saw-scaled viper) [12].

Tertiary medical facilities across these regions document high snakebite incidence rates, and their mortality levels depend on patients' access to medical facilities and antivenom supplies. Since the researchers examined Haryana over five years, they highlighted that reporting issues and inadequate healthcare monitoring systems have caused significant disease burdens to remain unknown [13]. The higher case fatality rates in Uttarakhand and Himachal Pradesh stem from both emergency care delays caused by difficult terrain and the shortage of skilled healthcare staff at primary healthcare facilities [14]. A localized public health strategy needs development to address snakebite-endemic zones because these areas have unique topographical, climatic, and infrastructural conditions [15].

Many cases of unidentified snakes result in generalized envenomation classification, which leads healthcare providers to administer non-specific treatment methods. Hospitals face challenges in distinguishing between hemotoxic and neurotoxic bites when diagnostic confirmation is absent, which leads to either excessive treatment or incorrect selection of ASV [16]. National health information systems require snakebite integration for improved policy development and targeted resource distribution to specific regions [17]. Table 1 presents comparative data regarding snakebite incidents and death rates, together with species distribution and healthcare access across major northern Indian states, to demonstrate how these states differ in terms of their response capabilities.

**Table 1:** Comparative epidemiological burden of hemotoxic snakebites in northern Indian states Table credits: Vikas Raj

State	Estimated Annual Cases	Case Fatality Rate (%)	Dominant Hemotoxic Species	Reporting Efficiency	Primary Healthcare Access (Rural)	References
Uttar Pradesh	35,000–40,000	4.2	Daboia russelii, Echis carinatus	Moderate	Low	[12,15]
Punjab	6,000–8,000	2.8	Daboia russelii	Low	Moderate	[13]
Haryana	7,000–9,000	3.5	Echis carinatus	Low	Moderate	[9]
Uttarakhand	3,000–4,500	5.1	Daboia russelii	Very Low	Low	[14]
Himachal Pradesh	2,500–3,500	6.0	Daboia russelii	Very Low	Very Low	[14]

Toxicological profile of hemotoxic venoms in the region

The biochemical profiles of northern Indian hemotoxic venoms reveal extensive diversity because they show both distinct venom properties between different species and venom variations within the same species that depend on geographical location. The procoagulant activity of Russell’s viper venom consists of serine proteases and metalloproteinases that cause uncontrolled blood clotting and fibrinogen breakdown, leading to disseminated intravascular coagulation. The venom from saw-scaled vipers attacks the vascular endothelium to cause bleeding inside the body and tissue death at specific locations [18]. The venom effects lead to the rapid and unpredictable clinical presentation of symptoms. The characteristic feature of venom-induced consumption coagulation (VICC) causes spontaneous bleeding, epistaxis, and hematemesis when left untreated. Advances in venom analysis through methods such as nanofractionation and proteomics gave scientists better control over understanding snake toxicities by showing biochemical differences in the same species across different regions [19]. The research shows that standard ASV administration methods need revision because they prove the necessity for localized antivenom formulations [20].

The venom composition in Northern India differs from that in Southern areas because existing ASVs were developed using venom primarily collected from snakes in Tamil Nadu [19]. This mismatch between venom composition and the ASV used for treatment results in reduced ASV effectiveness for northern snake species. The insufficient regional adaptation of antivenom affects patient outcomes when they follow treatment guidelines [21].

Clinical features and systemic complications of hemotoxic envenomation

Hemotoxic snakebite causes two groups of symptoms that need careful medical observation during assessment. The symptoms at the bite site include swelling with erythema, pain, and blister formation. The first stage of systemic involvement starts with coagulopathy, which healthcare providers detect through bedside clotting tests, including the 20-minute whole blood clotting time (WBCT). The condition advances to worse complications through hematuria and then hypotension before leading to acute kidney injury (AKI) [22]. Hemotoxic envenomation leads to acute kidney injury, which develops most often when patients receive delayed antivenom treatment. The development of AKI results from nephrotoxic damage along with microthrombi and hemoglobinuria caused by intravascular hemolysis. The research conducted in Myanmar showed that elevated serum creatinine levels, along with hematuria, served as robust indicators for renal involvement in snakebite patients, thus highlighting the importance of early medical intervention [23]. Children who experience snakebites face accelerated systemic disease progression, so they need specialized immediate medical attention [24].

**Figure 2:**
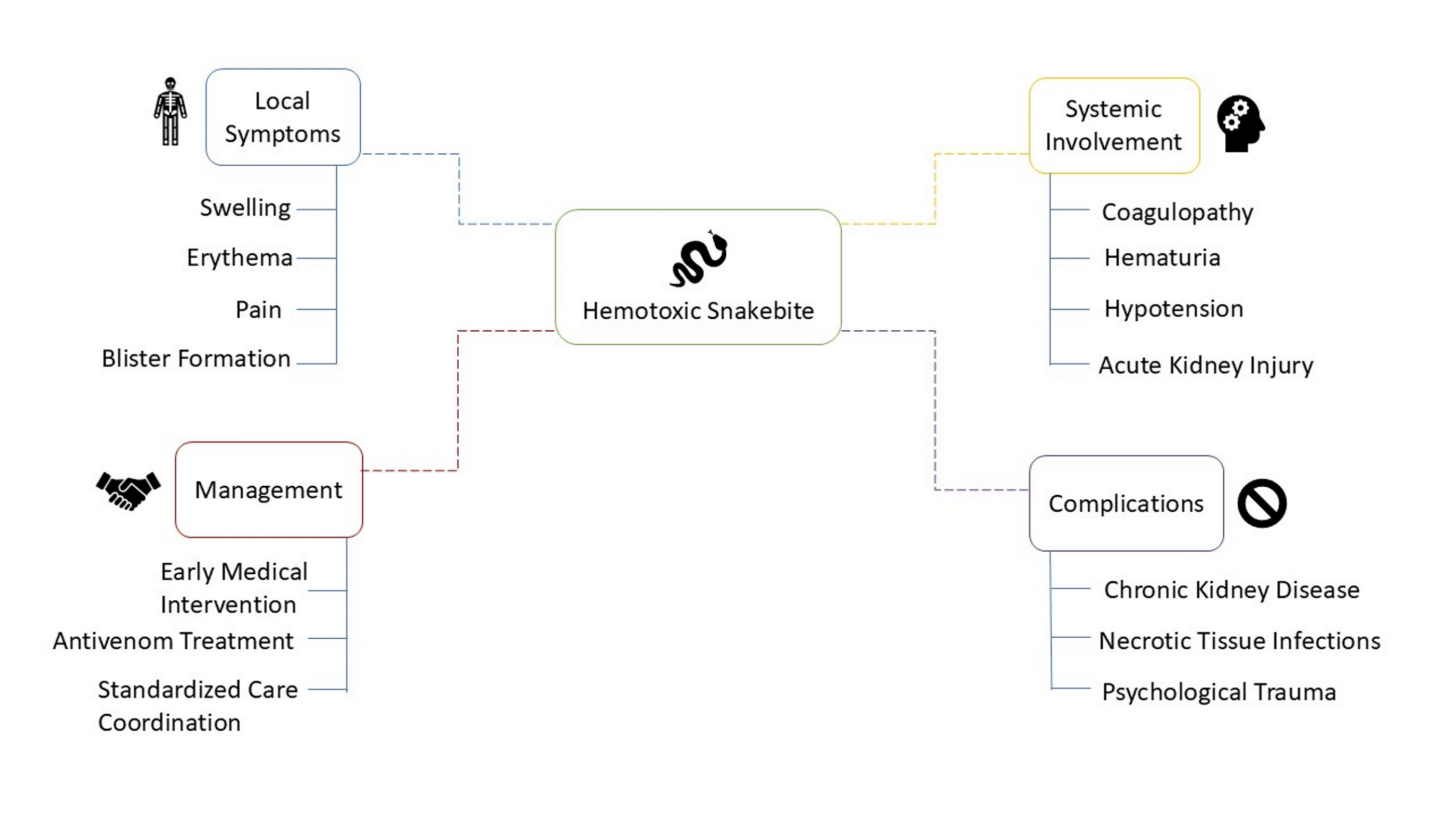
Hemotoxic snakebite: symptoms, complications, and management Image credit: Vikas Raj Figure created using Microsoft PowerPoint

Hemotoxic envenomation that remains untreated or receives delayed treatment leads to various long-term complications, including chronic kidney disease and necrotic tissue infections, along with psychological trauma [25]. Patients in specific areas face additional complications from unproven medical treatments that healthcare providers administer before hospital admission. The wide range of clinical manifestations requires prompt identification of patients along with standardized care coordination between multiple healthcare providers in emergency departments and post-acute care facilities [26]. Figure 2 illustrates the key symptoms, complications, and management strategies associated with hemotoxic snakebite, highlighting local and systemic effects along with essential treatment approaches.

National guidelines on ASV therapy: frameworks and shortcomings

The WHO, along with other national and global health bodies, provides standardized protocols for treating snakebite envenomation. The guidelines specify under which circumstances to use ASV, along with recommended starting doses and monitoring protocols. The WHO suggests using ASV after detecting systemic envenomation symptoms, which include spontaneous bleeding and neurological problems, and prolonged blood clotting times. The WHO recommends starting treatment of hemotoxic envenomation with an initial 8 to 10 vial dose and subsequent dosing based on clinical need [26].

The provided guidance for ASV management exists, but healthcare providers implement it with varying levels of consistency. Multiple Indian studies reveal that medical practitioners base their ASV dosing on personal experience and drug availability instead of established protocols [27]. Training and capacity building represent essential points in the National Action Plan, yet frontline workers struggle to implement protocols because they lack proper awareness and resources [28].

The existing national framework depends on a polyvalent antivenom that was developed against four specific species only. The current approach fails to recognize regional and uncommon venomous species that induce hemotoxicity in northern forest areas and rural settings. The absence of investments in venom collection from diverse geographic regions and ASV manufacturing will prevent national guidelines from effectively handling hemotoxic snakebite situations in northern India [29].

Interstate variation in ASV dosing protocols and practices

The absence of standard ASV dosing protocols throughout India leads healthcare facilities to follow various treatment approaches even when located in the same geographic area. The tertiary care facilities in Punjab and Haryana initiate ASV treatment with 10-20 vials before adjusting the dose according to clotting test results. Peripheral facilities tend to provide reduced ASV doses because they either lack sufficient supplies or want to minimize adverse effects [29]. Clinicians show inconsistent results because they depend solely on their professional experience when laboratory diagnostics are not available [30].

The survival results from a critical review showed no substantial differences between high-dose and low-dose administration when healthcare providers optimized their delivery methods and timing. The high-dose groups demonstrated higher rates of adverse effects that included anaphylaxis and serum sickness [31]. Various Indian and Southeast Asian studies agree that symptom progression should guide drug dosage adjustments instead of using standard fixed drug distribution [32]. 

The lack of standardized treatment methods has prompted medical organizations to introduce regional algorithms that incorporate venom-specific manifestations, service delivery factors, and patient demographics. Resource-limited settings require such protocols because they experience frequent stockouts and expired vials along with empirical ASV administration without follow-up testing. Adaptive treatment protocols developed locally would create better clinical results and protect scarce antivenom resources [15]. The data in Table 2 show that snakebite risk in Northern India depends on seasonal variations, occupational hazards, and patient behavior when seeking medical help. The table identifies the necessity for location-specific strategies that prevent and intervene against snakebites.

**Table 2:** Seasonal trends and exposure risk factors in snakebite-endemic states of northern India Table credits: Vikas Raj

State	Peak Snakebite Months	High-Risk Occupations	Community Exposure (%)	Average Daily Field Hours	Common Bite Locations on the Body	% Seeking Traditional Healers	Reference(s)
Punjab	June–August	Farmers, Field Laborers	65	6–8 hours	Feet, Lower Legs	28%	[21]
Haryana	June–September	Agricultural Workers	58	5–7 hours	Ankles, Hands	32%	[16]
Uttar Pradesh	July–September	Paddy Farmers	72	7–9 hours	Feet, Hands	40%	[12]
Uttarakhand	May–July	Forest Dwellers	50	4–6 hours	Legs, Arms	55%	[3]
Himachal Pradesh	May–June	Shepherds, Outdoor Workers	45	5–6 hours	Legs, Hands	47%	[24]

Importance of bite-to-needle interval in determining outcomes

The period between venomous snakebite and ASV treatment, known as the bite-to-needle interval, determines the patient outcomes in cases of hemotoxic envenomation. Research shows that starting ASV therapy in the first 4 to 6 hours after envenomation leads to reduced complications and death. When venom distribution reaches the vascular and lymphatic systems after the first 4 to 6 hours, it causes permanent endothelial damage and organ dysfunction along with coagulopathy [27].

Research conducted in the hilly regions of North India, specifically Himachal Pradesh and Uttarakhand, shows that the delayed time between snake bite and medical treatment leads to worsened patient outcomes, particularly renal complications and longer hospital stays [10]. The outcome of patients treated at tertiary hospitals in Punjab and Haryana deteriorates when they reach more than six hours post-bite because acute kidney injury and systemic bleeding occur more frequently, along with fatal cerebral hemorrhage [13].

Medical professionals have introduced bite-to-needle time extrapolation as a potential method to predict or categorize treatment results based on this measurement interval. Rural clinicians should begin administering ASV therapy by empirical means when snakebite is suspected, and envenomation signs appear in patients before all laboratory tests become available [3]. The public and community health workers need proper education about early treatment referral because it shortens the time to care and improves survival chances.

Route of ASV administration: clinical impact and logistical realities

The method through which ASV reaches the body directly influences how the drug performs and how it affects patient safety, as well as its distribution throughout the body. Medical professionals consider intravenous (IV) delivery the most efficient method because it enables quick venom toxin removal from the bloodstream [33]. The practice of intramuscular (IM) delivery remains a secondary method for venomous snakebite treatment since it produces delayed absorption and reduced drug availability.

Medical research shows that ASV administered through the intravenous route leads to accelerated coagulation profile recovery and reduces systemic complications, mainly in patients experiencing moderate-to-severe hemotoxic envenomation [34]. The need for intramuscular administration arises in pediatric cases and rural centers with limited personnel, but healthcare providers perform this procedure with reluctance.

**Figure 3:**
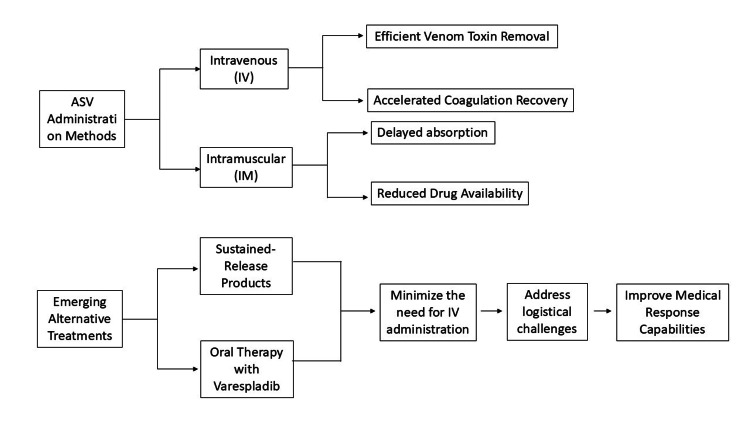
ASV administration methods and research advancements Image credit: Vikas Raj Figure created using Microsoft PowerPoint

Current scientific investigations focus on two unique delivery systems that combine sustained-release products along with oral therapy through varespladib to combat venom phospholipase A2 activity, thereby minimizing the need for IV administration [35]. The emerging alternative treatment methods show promise for future implementation because they could help solve the logistical challenges that affect North Indian medical response capabilities [21]. The selection between different administration paths depends on both the need for swift interventions and the practical options available within the given medical environment. The current standard of IV administration remains essential, but healthcare providers should develop guidelines that enable early medication delivery through any available approach and subsequent patient transfer to advanced medical facilities. Figure 3 depicts the comparative approaches for ASV administration: intravenous (IV), intramuscular (IM), and emerging alternatives like oral therapy with varespladib.

ASV-induced adverse reactions: spectrum, risk, and management

The medical use of ASV presents several risks because adverse drug reactions continue to make treatment choices more complex. Adverse reactions to ASV treatment range from mild allergic responses such as urticaria to severe manifestations, including anaphylactic shock, bronchospasm, and serum sickness. The observed incidence rates for adverse drug reactions following antivenom administration span from 10% up to 80%, according to antivenom formulation, patient immunogenic profile, and rate of administration [36].

Patient safety faces significant risk in rural North Indian hospitals because these facilities lack emergency equipment such as adrenaline auto-injectors or ventilators during hypersensitivity reactions. The risk of hypersensitivity reactions increases when ASV administration occurs multiple times or when higher dosages are used, particularly when bite-to-needle time becomes extended [11]. The practice of using slower infusion rates or premedicating with antihistamines and corticosteroids shows uncertain effectiveness [32].

Equine-derived ASV immunogenicity continues to be a significant challenge because researchers are now focused on developing purification techniques and humanized or recombinant antivenoms with lower hypersensitivity risks [33]. Present-day risk mitigation mostly relies on fast diagnosis of allergic reactions, followed by immediate epinephrine administration with supplementary treatment. Every healthcare facility delivering ASV therapy needs appropriate resuscitation equipment and staff members who have received proper training.

The evaluation of ASV benefits against potential adverse drug reactions is now a priority for clinicians when treating mild venomous cases. People experiencing different risks from treatment complications should use specific clinical and historical factors when determining the best way to maximize outcomes safely.

Healthcare system readiness and its role in treatment outcomes

The ability of healthcare systems to handle snakebite emergencies serves as a critical factor that influences treatment results, especially in locations where hemotoxic envenomation occurs frequently. Complex envenomation cases receive inadequate medical care in most rural and semi-urban areas across northern India. Inadequate ASV supply, combined with untrained staff and minimal laboratory and dialysis facilities, leads to delayed or incorrect medical treatment [13].

The connection between primary health systems and secondary or tertiary care providers is fragmented, leading to delays in critical treatment. Patients in Uttarakhand and Himachal Pradesh need to travel multiple hours by walking or using dangerous mountain roads to reach their first medical care facilities. During the monsoon season, hospitals in Punjab and Haryana experience temporary shortages because of increased bite cases [15].

Failure to maintain cold-chain requirements during ASV storage reduces vial effectiveness before remote centers receive them [14]. The National Health Mission utilizes decentralization of stock distribution and telemedicine consultation promotion to address these issues, yet their implementation remains inconsistent across regions [12]. Systemic capacity-building is thus essential. Point-of-care diagnostic tools and standardized treatment kits with resuscitation drugs should be provided to healthcare workers, while regular simulation training should be conducted for primary healthcare staff. The combination of healthcare integration with systems that guarantee availability, accessibility, and accountability will boost snakebite treatment results for vulnerable populations.

Table 3 demonstrates substantial differences in Northern Indian states regarding their infrastructure and workforce preparedness to treat snakebites through their levels of cold chain maintenance and availability of ASVs and trained medical staff, and operational treatment facilities. The deficiencies in these systems negatively affect both the speed and standard of medical assistance delivered during snakebite incidents.

**Table 3:** Critical infrastructure and workforce capacity for snakebite management in northern Indian states Table credits: Vikas Raj

State	Districts with Cold Chain Facilities	Districts with 24x7 ASV Availability	Avg. ASV Stockout Duration (days)	Functional Snakebite Treatment Units	Availability of Trained ASV Staff (%)	Reference(s)
Punjab	12	8	3	10	68	[34]
Haryana	9	6	4	7	61	[15]
Uttar Pradesh	15	10	5	12	70	[4]
Uttarakhand	5	3	7	4	49	[25]
Himachal Pradesh	3	2	9	2	42	[17]

Host-specific variables influencing ASV effectiveness

The therapeutic outcomes of ASV depend heavily on multiple variables that exist within the host. Various patient characteristics, including age and body weight, the presence of comorbidities and hydration status, and immune response, affect the distribution and metabolism and treatment effectiveness of ASV. Children face severe systemic complications from small amounts because their blood volume is lower, so medical staff must provide timely and precise dosing [24].

The appropriate administration of ASV does not guarantee safe outcomes for older adults because their existing health conditions, such as diabetes, hypertension, and renal impairment, increase the risk of complications [23]. People who have received equine serum through past treatments or bites face increased sensitivity to the substance, which demands special protocols for administering ASV. The location of the snake bite determines how venom spreads through the body, which affects symptom progression and the timing of ASV treatment effectiveness [34].

The research demonstrates why healthcare providers should individualize ASV therapy instead of following strict standard procedures. The variability of human hosts requires that clinical decisions to be based on their decisions. On early assessments, which inform dosage levels and infusion rates, and additional treatment needs [37]. A patient-specific ASV recommendation system will be feasible after developing predictive models that include both biometric and clinical variables for assessment.

Clinical grading and assessment tools in ASV decision-making

The success of ASV treatment relies on both having ASV available and performing precise clinical evaluations and proper patient prioritization. The clinical grading systems function as essential instruments for determining envenomation severity levels, which helps medical professionals determine appropriate treatment doses. The signs of prolonged clotting time and hypotension alongside spontaneous bleeding and laboratory results showing thrombocytopenia and elevated serum creatinine indicate the need to start or increase ASV treatment in hemotoxic cases [22].

Healthcare providers in rural northern Indian health settings must resort to making empirical decisions because laboratory support is unavailable in many locations. Medical staff must depend on clinical signs for treatment, yet this practice results in inconsistent medication amounts and sometimes improper levels of care [27]. Various Indian research studies push for developing basic severity assessment tools that healthcare providers can use at a patient's bedside. The implementation of standardized scoring systems allows healthcare providers to classify patients into mild, moderate, and severe envenomation categories so they can administer ASV treatment based on the appropriate tier [17].

The addition of mobile diagnostic technologies alongside point-of-care tests for coagulation would help clinicians make evidence-based ASV dosages. Tertiary centers require this approach most during peak seasons because they need to prioritize resources effectively when patient flow reaches its highest point. Simple monitoring methods like serial 20-minute WBCTs, along with vital parameter measurements, enable healthcare providers to establish safe ASV practice in diagnostic environments with limited capabilities.

Comparative perspectives on high- and low-dose ASV protocols

The Indian population engages in extensive discussions about the effectiveness and safety levels between high-dose and low-dose ASV regimens. Hemotoxic snakebites require particular attention because of the venom amount, the time between bite and treatment, and the patient's health condition determines the severity of symptoms. High-dose ASV administration is commonly used for severe systemic conditions and delayed envenomation, yet it carries the disadvantage of triggering hypersensitivity reactions [31].

Low-dose treatment approaches are now preferred because they show similar treatment results in moderate conditions, yet present better safety characteristics. The multicenter randomized controlled trial demonstrated that the controlled administration of 10-15 vials of ASV successfully reversed coagulopathy in most patients suffering from hemotoxic bites [30]. A systematic review found that, combined with proper clinical monitoring and dedicated doses of treatment, this approach enabled a practical and cost-effective approach to managing snake bites [38].

The current policy may not work for every patient with its set dosage. Different healthcare providers support an approach that adjusts medication doses according to repeated clinical evaluations of individual patient characteristics, along with modifications in their symptoms. The approach holds special importance in situations with restricted ASV supplies or complicated adverse reactions because of insufficient intensive care facilities [29]. Multiple experts now agree that clinical judgment combined with patient classification and hospital capability levels should determine the final dosage method.

Barriers in ASV supply and regional access inequities

The inconsistent distribution of ASV throughout Indian states and districts continues to be a major challenge for snakebite management. The essential drug ASV remains unavailable at every healthcare level, especially throughout remote and poorly funded regions of northern India. The monsoon season regularly brings periodic shortages of anti-snake venom, which leads to preventable complications and mortality [13].

The ASV products used in India mainly originate from venom obtained in southern states, which creates a potential issue of venom-antivenom incompatibility between regions. The clinical and proteomic evidence indicates that antivenom-venom mismatches decrease northern snake species' treatment response despite appropriate dosing [21]. The quality of ASV decreases when peripheral centers experience improper cold chain maintenance and inconsistent inventory control [11].

Hospital facilities throughout Punjab, Haryana, and Uttar Pradesh have started limiting access to ASV due to unreliable supply chain systems, which compel doctors to determine which patients will receive complete treatment. The national programs have launched decentralization schemes and tracking systems to combat these problems, yet their implementation remains partial and inconsistent between districts [12]. Early-phase trials of metalloproteinase inhibitors and phospholipase A2 antagonist varespladib show promising results [35]. These new agents are currently under development for potential use in reducing ASV dependence and providing temporary relief when stockouts occur. The main priority remains to fix both supply chain and logistical issues until these new treatment methods become commonly available. The implementation of state-level ASV banks and data-driven distribution systems alongside continuous availability in high-risk blocks constitutes essential measures to address the issue.

Strengthening public health infrastructure and training for snakebite response

The outcomes of snakebites depend equally on vital clinical and pharmaceutical treatments, along with the broader public health infrastructure and training systems. The implementation of the National Action Plan for Snakebite Envenoming (NAPSE), along with WHO-supported guidelines, encounters obstacles at the field level because of limited resources and bureaucratic delays [39].

The insufficient training of primary healthcare providers stands as a significant barrier to proper snakebite treatment in rural and sub-district hospitals. Research indicates that doctors and nurses working in high-incidence areas lack formal training about snakebite management and ASV administration [39]. The result of this situation causes healthcare providers to use trial-and-error methods while patients experience delayed medical attention and receive inadequate drug administrations. Research indicates that simulation-based workshops, along with mobile training apps and visual treatment algorithms, help providers develop better competence and confidence. The earliest successful reaction to snakebites depends heavily on public awareness initiatives. Many areas throughout Northern India continue to use incorrect first-aid methods, such as tourniquets and incisions, and herbal remedies, which harm victims while delaying professional treatment [27]. The implementation of community outreach programs, which include Accredited Social Health Activists (ASHAs) and school awareness programs, and local leader engagement will help reduce these harmful practices.

Infrastructure-wise, many centers still lack necessities such as functional blood coagulation analyzers, dialysis machines, or even oxygen supplies. Snakebite management integration within the Health and Wellness Centers established under Ayushman Bharat could result in sustained improvements across the healthcare system. National digital health surveillance systems that track snakebite data will benefit policymakers by letting them identify high-risk areas while enabling them to optimize ASV logistics processes. A comprehensive solution that unites medical protocols with staff training while involving communities, along with health system reforms, will help address multiple obstacles that maintain snakebite as a severe public health problem. Table 4 shows that major training gaps between frontline health workers in snakebite management cause both clinical mistakes and substandard patient outcomes. Targeted educational programs need to address these poor outcomes caused by healthcare worker training gaps to enhance survival rates and treatment accuracy in rural healthcare facilities.

**Table 4:** Training deficits and field-level practice gaps in snakebite management Table credits: Vikas Raj WBCT: Whole blood clotting time

Training Area	Untrained Workers (%)	Reported Errors in Field Cases (%)	Impact on Patient Outcome	References
Identification of Hemotoxic vs. Neurotoxic Bites	62	48	Delayed or incorrect ASV type used	[40]
Correct Dose Escalation Strategy	70	53	Under- or over-dosing of ASV	[21]
Recognition of Adverse Drug Reactions	65	51	Adverse effects go unnoticed or unmanaged	[2,7]
Emergency Management of Anaphylaxis	74	60	Delayed adrenaline administration, higher mortality risk	[35]
Use of WBCT (20-min clot test)	68	56	Inaccurate envenomation grading and ASV misuse	[14]

Methodological considerations and limitations

This research analyzed optimal ASV dosing and clinical results in northern Indian hemotoxic snakebite patients through a synthesis of peer-reviewed literature, clinical studies, and policy reports. The review team selected literature based on its geographical relevance and strong methodologies, as well as its clinical focus on hemotoxic envenomation that specifically involved *Daboia russelii* and *Echis carinatus*. The research utilized the keywords “hemotoxic snakebite,” “anti-snake venom dose,” “ASV complications,” “coagulopathy,” “acute kidney injury,” “Russell’s viper,” “saw-scaled viper,” “India,” and “snakebite outcomes” in the search strategy. The research utilized these keywords for database searches in PubMed, Google Scholar, Scopus, and regional journals. The research method allowed investigators to grasp comprehensive epidemiological data as well as treatment differences and clinical obstacles across various healthcare environments. The synthesis utilized primarily retrospective studies and observational research and case series, but most of these publications lacked standard measures, patient grouping, and treatment protocols. The research limitations create challenges for finding comparisons between studies and weaken the overall strength of clinical recommendations. Even so, the review left out pertinent information contained within unpublished datasets, community health records, and grey literature, including underreported rural and tribal regions. The majority of studies originated from single-center facilities or hospitals, which reduces their ability to represent the wider population [7]. The emphasis on hemotoxic envenomation restricts the practical use of findings to locations with neurotoxic or mixed envenomation characteristics. The literature lacks comprehensive information about two emerging treatment options, which include varespladib and recombinant antivenoms, because these treatments remain in the clinical investigation stages. Traditional limitations in research studies underline the necessity for multicenter prospective investigations that use standard methods to create evidence-based and location-specific antivenom strategies.

Future directions

Research on snakebite treatment in northern India should use multiple medical centers to test standard ASV doses that match local venom types. Research must create region-specific antivenoms that match venom differences between snakes in each area. Scientists should test oral drug varespladib and new antivenom products along with monoclonal antibodies through well-designed studies to check their safety and effectiveness. Accurate monitoring systems plus electronic health record sharing will help us identify danger areas based on location data [31]. The public health system needs funds for building safe clinics, plus nurse and patient training, with awareness promotion to provide better early treatment and save more lives. Working together between medical staff and experts in both toxicology and health administration enables research results to benefit society. Studies that show how much an intervention costs and research done with local people will help us create programs that work well for rural patients who need snakebite care.

## Conclusions

Snakebite envenomation causes severe health problems in India that people often ignore, especially in the northern states of Punjab, Haryana, Uttar Pradesh, Uttarakhand, and Himachal Pradesh. Hemotoxic snakes, *Daboia russelii* and *Echis carinatus*, cause many deaths and illnesses because they damage blood clotting and lead to bleeding inside the body and kidney failure. This review examined multiple studies to determine the most effective ASV treatment methods, revealing that varying medical approaches led to different results. Research now shows that low-dose snake venom treatment works well when given early and watched closely, even though doctors still use high doses for serious cases. How patients respond to treatment depends on different aspects, such as their health, how venom moves through the body, where ASV is given, and how much ASV is available.

The review shows that healthcare systems must be ready to treat snakebites effectively, while workers need proper training and better patient transfer options to give ASV on time. Research shows that new oral medicine varespladib and monoclonal antivenom products might solve current treatment problems, but need more testing and validation. A future approach to snakebite management should combine local evidence with medical expertise and public health knowledge. Better policy support, combined with public education and local healthcare services, will decrease the number of people who die or suffer from snakebites.
